# Electrochemical Trimethylamine *N*-Oxide Biosensor with Enzyme-Based Oxygen-Scavenging Membrane for Long-Term Operation under Ambient Air

**DOI:** 10.3390/bios11040098

**Published:** 2021-03-27

**Authors:** Armel F. T. Waffo, Biljana Mitrova, Kim Tiedemann, Chantal Iobbi-Nivol, Silke Leimkühler, Ulla Wollenberger

**Affiliations:** 1 Institute for Biochemistry and Biology, University Potsdam, 14476 Potsdam, Germany; tadjoungwaffo@tu-berlin.de (A.F.T.W.); b.mitrova@gmail.com (B.M.); kim.tiedemann@uni-potsdam.de (K.T.); sleim@uni-potsdam.de (S.L.); 2 CNRS, Aix-Marseille Université, Institut de Microbiologie de la Méditerranée, Laboratoire de Bioénergétique et Ingénierie des Protéines, 13402 Marseille, France; iobbi@imm.cnrs.fr

**Keywords:** trimethylamine *N*-oxide, biosensor, TMAO-reductase, oxygen scavenger, immobilized enzyme, multienzyme electrode, viologen

## Abstract

An amperometric trimethylamine *N*-oxide (TMAO) biosensor is reported, where TMAO reductase (TorA) and glucose oxidase (GOD) and catalase (Cat) were immobilized on the electrode surface, enabling measurements of mediated enzymatic TMAO reduction at low potential under ambient air conditions. The oxygen anti-interference membrane composed of GOD, Cat and polyvinyl alcohol (PVA) hydrogel, together with glucose concentration, was optimized until the O_2_ reduction current of a Clark-type electrode was completely suppressed for at least 3 h. For the preparation of the TMAO biosensor, *Escherichia coli* TorA was purified under anaerobic conditions and immobilized on the surface of a carbon electrode and covered by the optimized O_2_ scavenging membrane. The TMAO sensor operates at a potential of −0.8 V vs. Ag/AgCl (1 M KCl), where the reduction of methylviologen (MV) is recorded. The sensor signal depends linearly on TMAO concentrations between 2 µM and 15 mM, with a sensitivity of 2.75 ± 1.7 µA/mM. The developed biosensor is characterized by a response time of about 33 s and an operational stability over 3 weeks. Furthermore, measurements of TMAO concentration were performed in 10% human serum, where the lowest detectable concentration is of 10 µM TMAO.

## 1. Introduction

Cardiovascular diseases (CVDs) are the major cause of death globally. Over the past decades, public health experts have found a role of the gut microbiome in connection with the diet behavior in the development of such diseases. For instance, the human microbiota consisting of more than 10^14^ microorganisms, with a very big diversity, is known to affect the whole human body through the production of metabolic compounds from dietary contents [1].

Trimethylamine *N*-oxide (TMAO) is the product of the degradation of nutrients rich in trimethylamine (TMA)-containing compounds such as choline, carnitine and lecithin by gut microbiota [2]. Recent studies associated TMAO with the risk of developing complex diseases, including CVDs [3,4,5], colorectal cancer [6], chronic kidney diseases [5] diabetes [7] and obesity [8]. Therefore, TMAO is emerging as a relevant biomarker for the aforementioned diseases, and its analytical monitoring is thus important in health management.

Established methods require expensive laboratory equipment and/or skilled personnel and are therefore restricted to big laboratories [9]. These methods are chromatography-based methods such as gas chromatography-mass spectrometry (GC-MS) [10], flow injection gas diffusion-ion chromatography (FIGD-IC) [11], high-performance liquid chromatography (HPLC) [12] and ultra-high performance liquid chromatography-tandem mass spectrometry (UPLC-MS) [13]. Electrochemical biosensors represent an attractive alternative as analytical methods, in particular for the decentralized tests in small laboratories or on-site monitoring, as they offer a fast response time and high selectivity, as well as the potential for low-cost simple operation in miniaturized devices [14]. To the best of our knowledge only three publications report sensors for TMAO, i.e., a microbial sensor using *Pseudomonas aminovarans* on an oxygen electrode [15], a sensor with molecularly imprinted polymers on ITO [16] and an enzyme biosensor with an enzyme immobilized on a glassy carbon electrode [17].

The TMAO reductase (TorA, EC 1.7.2.3) is a mononuclear molybdenum-containing enzyme from the DMSO reductase family. This enzyme catalyzes the reduction of TMAO by two electrons and two protons into TMA and water [18], as shown in Equation (1).

TMAO + 2H^+^ +2e^−^ → TMA + H_2_O (1)

TorA also converts other *N*-oxide compounds such as methylmorphine-*N*-oxide, picoline-*N*-oxide, chloropyridine *N*-oxide and nicotine *N*-oxide, but catalyzes TMAO with the highest efficiency [19]. Furthermore, from all the above-mentioned *N*-oxide compounds, only TMAO is naturally present as a metabolic product in humans, thus making TorA well suitable for biosensor application in diagnostics.

Previously, we reported an electrochemical biosensor for the determination of TMAO levels in human serum [17]. The TMAO biosensor was based on an enzymatic reaction where TMAO could be detected through its catalytic reduction in the presence of a chimeric TMAO reductase (TorA-FDH): TorA variant reconstituted with the molybdenum cofactor from a formate dehydrogenase (FDH) [20]). This specifically produced TMAO reductase was immobilized on the surface of a glassy carbon electrode (GCE) and held in place by a dialysis membrane. The electrochemical reduction of methyl viologen, (MV, mediator) present in the solution, generated the methyl viologen cation radical, as shown in Equation (2), which provided to TorA-FDH the electrons needed for the reduction of TMAO into TMA (Equation (1)). The net reaction involving MV and TMAO reduction is described by Equation (3).

MV^2+^ + e^−^ ↔ MV^+●^ (2)

TMAO + 2H^+^ + 2MV^+●^ → TMA + 2MV^2+^ + H_2_O (3)

The mediated electrocatalytic TorA reaction is susceptible to oxygen disturbance, and therefore attempts had to be made to remove oxygen. This problem is common to all biosensors based on reductases. The presence of O_2_ interferes with the detection of the analyte because oxygen reduction occurs also at the potential required for the mediator reduction [21]. The electrochemical reduction of oxygen generates a large cathodic current which overlaps with the analytical signal of mediator reduction. For this reason, a high background current is usually observed in the presence of oxygen. Higher background currents influence the quality of the system, since the noise is typically much higher, and therefore the error of the measurements increases, i.e., the biosensor reproducibility and sensitivity decrease. In addition, the incomplete electrochemical reduction of O_2_ also produces reactive oxygen species (ROS) that might be deleterious to the biorecognition element [22]. Furthermore, reduced low-potential mediators react with dissolved O_2_ and ROS [23]. Viologens are examples of mediators used in reductase-based biosensors. Formal potentials of viologens are generally lower than those of O_2_ and their incomplete reduction in aerobic conditions leads to the production of ROS [21]. In order to overcome these limitations, standard oxygen removal methods employ argon/nitrogen purging and vacuum degassing which are not suitable for the simple setups typically used for enzyme electrode measurements. Alternative oxygen elimination methods employ coupled enzyme reactions such as GOD and Cat in the presence of glucose to transform the dissolved molecular oxygen into water. These are very efficient in ensuring anoxic conditions in biochemical experiments in solution. The GOD/Cat/glucose approach was applied to reduce the oxygen in biosensors based on nitrate reductase, nitrite reductase [21,24] and DMSO reductase [25]. Moreover, for the above-mentioned chimeric TorA-based biosensor, GOD and Cat were added to the measuring solution containing glucose to degrade the oxygen in the solution enzymatically [17]. However, for the practical use of biosensors additional solution components and long waiting times are undesired, and therefore oxygen scavenging membranes were applied which assure local oxygen depletion on the electrode surface for nitrite and DMSO measurement [21,26,27].

**Scheme 1:**
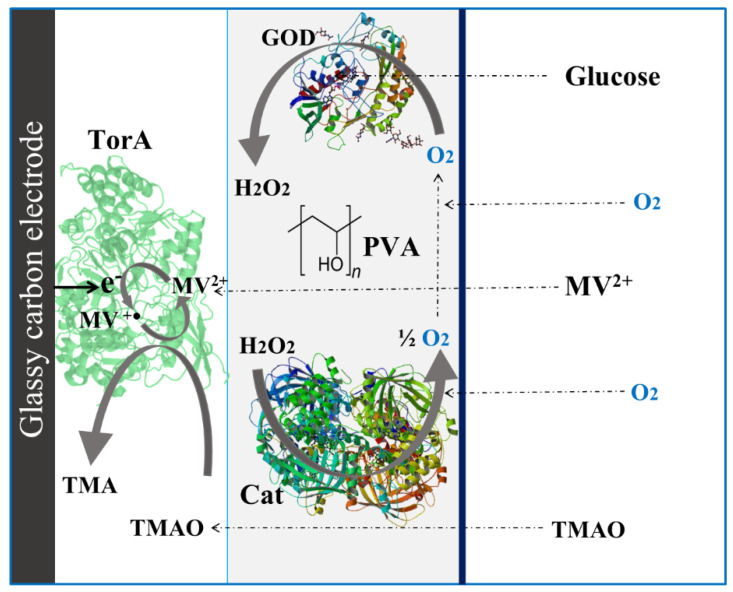
Illustration of the trimethylamine *N*-oxide (TMAO) biosensor with its different components.

In this work, we have developed an electrochemical TMAO biosensor using immobilized *E. coli* TMAO reductase (TorA), which senses TMAO in the presence of the low potential mediator MV. The cathodic reduction of the mediator is ensuring the regeneration of the enzyme at the electrode surface and results in a measurable signal. GOD and Cat were immobilized on the electrode surface in a second layer to remove oxygen enzymatically from the electrode surface while the oxygen in the bulk solution may still be present, and thus to eliminate the oxygen background current and to protect the immobilized TorA from inactivation (Scheme 1). We optimized the O_2_ scavenging membrane to maintain anaerobic conditions for a longer time period. The mediated electrochemistry of TorA using MV proved to be suitable for the detection of low TMAO concentrations in human serum samples. The immobilization of an O_2_ scavenging membrane together with TorA on GCEs represents a step forward in the development of a disposable analytical tool for monitoring TMAO.

## 2. Materials and Methods

### 2.1. Reagents and Solutions

TMAO and MV were purchased from Sigma-Aldrich (Darmstadt, Germany), and stock solutions were prepared with deionized water (18.2 MΩ cm) from the Millipore Milli-Q purification system. Potassium dihydrogen phosphate, tris(hydroxylmethyl)aminomethane (TRIS), disodium hydrogen phosphate, potassium chloride and glucose were purchased from Merck. Polyvinyl alcohol (PVA) (MW = 22,000 g/mol) was purchased from SERVA. The Alpha A alumina powder (aluminum oxide 0.3 and 1 µm) used for the cleaning of the glassy carbon electrode (GCE) was purchased from Leco (Geleen, The Netherlands). Cat from bovine liver (10,000 U/mg) and GOD from *Aspergillus niger* (149.5 U/mg) were purchased as lyophilized powder from Sigma and used without any further purification. The human serum was purchased from SERO AS, Norway. The dialysis membrane (MWCO 9 kDa) and the polyethylene membrane (PE) were from BST Biosensor Technology (Berlin, Germany). The *E. coli* TorA (559.23 U/mg, kcat 860.34 s^−1^) used in this work was anaerobically expressed and purified as reported elsewhere [20].

### 2.2. Apparatus and Procedure

#### 2.2.1. Oxygen Scavenging Membrane Preparation and Optimization

For the membrane preparation, 0.1 g of PVA were added to 1 mL of milli-Q water and heated for about 30 s until the complete dissolution of PVA. The obtained solution was stored at room temperature. New solutions were prepared after every week. GOD and Cat were dissolved in 50 mM sodium dihydrogen phosphate solution, pH 7, and mixed with the previously prepared PVA according to the needed concentration. The prepared solution containing PVA (3 to 5%) and the respective amounts of enzymes were then dropcasted onto a PE membrane or directly onto the electrode surface of GCE and placed under UV-light for a period of 15 min for PVA to crosslink. The oxygen scavenging membrane was stored at 4 °C.

The efficiency of the O_2_ scavenging membrane in removing O_2_ from the sensing layer was optimized by varying the concentration of each of its components (GOD, Cat and PVA). A Clark-type electrode composed of a platinum electrode (working electrode) and a silver/silver chloride electrode (Ag/AgCl: reference electrode and 1 M KCl electrolyte) was used for the simultaneous amperometric measurement of the O_2_ present in the sample as well as the H_2_O_2_ produced by the GOD catalyzed reaction. Prior to these measurements, the O_2_ scavenging membrane was sandwiched between two membranes using two different O-rings. Depending on whether H_2_O_2_ or O_2_ were being indicated, the O_2_ scavenging membrane was sandwiched between dialysis membranes or a dialysis and a PE membrane for the measurement of H_2_O_2_ and/or O_2_, respectively. The electrochemical measurements were performed at room temperature using Palmsens potentiostat and its PSLite 4.6 software (Palmsens, Houten, The Netherlands). The amperometric measurement was performed at different applied potentials (−0.6 V for O_2_ and +0.6 V for H_2_O_2_) and stirring of the solution.

#### 2.2.2. TMAO Biosensor

For biosensor preparation, the glassy carbon electrode (GCE, BAS) was polished with two different grain-sized alumina powders (1 and 0.3 µm), respectively. The cleaned GCEs were then ultrasonicated in acetone for 3 min and for 7 min in Milli-Q water. After drying under N_2_ stream, 5 μL of TorA were dropcasted onto the electrodes and incubated for 30 min in a desiccator at 4 °C. The modified electrodes were then covered with the previous optimized oxygen scavenging membrane hold by a dialysis membrane and placed in the electrochemical cell with an O-ring assembly as described elsewhere [17].

The electrochemical experiments were carried out in a lab-made three-electrode cell equipped with the GCE as working electrode, an Ag/AgCl reference electrode (Microelectrodes Inc., Bedford, MA, USA) and a Pt wire as counter electrode. The amperometric measurement was performed with an applied working potential of −0.8 V. The TMAO sensor was challenged with different concentrations of TMAO in the Sörensen phosphate buffer solution, pH 7, with 100 mM KCl, and in 1:10 diluted human serum in the same buffer solution.

All electrochemical measurements were performed at room temperature in laboratory condition using Palmsens potentiostats and analyzed with the PSLite 4.6 software (PAlmsens, Houten, The Netherlands) and OriginPro 2019.

## 3. Results and Discussion

### 3.1. Optimization of the Oxygen-Scavenging Membrane

In a first step we searched for conditions to optimize the anaerobic surrounding of the TorA enzyme trapped on the electrode. For this purpose, the sequential reaction of glucose oxidation and peroxide disproportionation was employed in a layer of coimmobilized GOD and Cat to deplete oxygen and destroy the produced hydrogen peroxide on the surface of the electrode. For an efficient oxygen depletion system, several requirements had to be fulfilled. A high GOD activity in the top layer is needed to ensure the total consumption of the oxygen diffusing from the bulk solution in the layer in front of the electrode surface, and enzyme excess is needed to counterbalance the inactivation of enzymes over time for a long-term use of the oxygen-scavenging property. Moreover, a sufficiently high activity of immobilized Cat is essential to destroy hydrogen peroxide. The maximum turnover is achieved by the addition of a saturation concentration of glucose. Finally, the polymer concentration determines the capacity to entrap enzyme molecules and influences the characteristic diffusion time.

The ability of the O_2_-scavenging membrane to ensure anoxic conditions on the sensor surface was first optimized with respect to the concentration of the GOD entrapped in a PVA hydrogel in the absence of Cat by recording the cathodic oxygen reduction in the dependence of glucose. The cyclic voltammogram (CV) displays a broad cathodic wave due to the electrochemical O_2_ reduction at a potential lower than −0.3 V in the absence of glucose (Figure S1a). The reduction wave in the cyclic voltammogram decreases immediately upon addition of glucose to the bulk buffer solution according to the GOD catalyzed glucose oxidation, as shown in Equation (4).

O_2_ + C_6_H_12_O_6_ → C_6_H_10_O_6_ + H_2_O_2_ (4)

H_2_O_2_ → ½ O_2_ + H_2_O (5)

In the amperometric measurement performed at a working potential of −0.6 V vs. Ag/AgCl, 1 M KCl, a current of about −1 µA was observed in the plain phosphate buffer solution (Figure S1b). The addition of 3 mM glucose resulted in a decrease of the reduction current as a result of the oxygen consumption in the membrane (Figure S1b). In parallel, the H_2_O_2_ formed due to the oxidation of the glucose was recorded at the potential of +0.6 V vs. Ag/AgCl 1 M KCl at a second sensor (Figure S1c). The GOD-loading of the electrode was varied, and 10 U GOD per membrane in the presence of 3 mM glucose (the value at which saturation is reached) were found to be sufficient to maintain anoxic conditions in the vicinity of the electrode (Figure S1d).

In the next step different concentrations, from 300 to 1000 U, of Cat were coimmobilized with 10 U GOD in 3% PVA in order to remove the formed H_2_O_2_ (Equation (5)).

**Figure 1:**
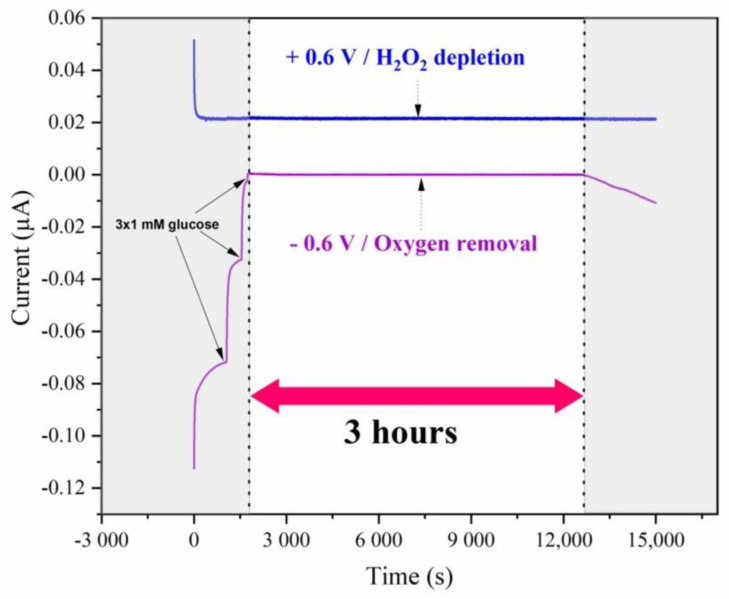
Performance of the scavenging membrane containing 10 U GOD and 1000 U Cat in 3% PVA. Current traces of the Clark electrode polarized at −0.6 V (purple curve: O_2_ removal) and +0.6 V (blue curve: H_2_O_2_ monitoring) after three additions of 1 mM glucose each. Measurements were performed under ambient air conditions in a Sörensen buffer solution (pH 7) containing 100 mM KCl.

Amperometric measurements were performed to analyze the efficiency of this membrane in removing the dissolved O_2_ without liberating a detectable amount of H_2_O_2_ at the surface of the electrode. As expected, and similarly to the previous experiments, the current of the oxygen sensor decreased upon addition of glucose (E_app_ = −0.6 V), and no current changes were recorded on the anode (E_app_ = +0.6 V). These observations demonstrate that the H_2_O_2_ produced in the membrane by GOD could be completely trapped by Cat (Figure 1). In conclusion, a scavenging membrane of 10 U GOD and 1000 U Cat entrapped in 3% PVA provides anoxic conditions without the detection of any produced H_2_O_2_ for at least 3 h on Clark electrodes due to the high turnover of GOD and Cat. These slight modifications of previously published conditions [21,24] led to an improvement of the oxygen depletion system since the two-enzyme system used in this study can operate without disturbance of O_2_ for 3 h instead of 2 h at a six times lower glucose concentration, i.e., 3 mM compared to 20 mM [21].

The O_2_ scavenging membrane also functioned when it was placed on a glassy carbon electrode (GCE), where anoxic conditions were maintained at the surface over a period of 3 h (Figure S2).

### 3.2. TMAO Biosensor

#### 3.2.1. Assembly of a TMAO Biosensor

**Figure 2:**
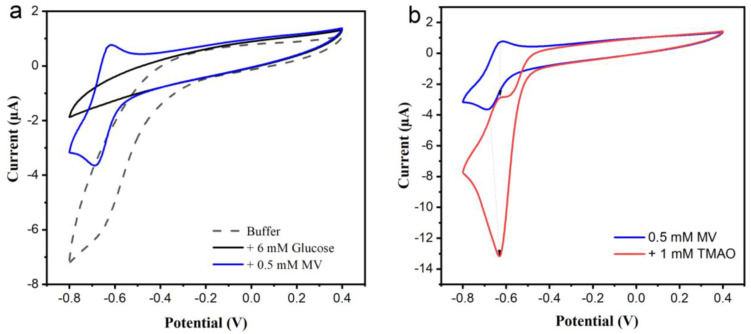
CV of TMAO reductase (TorA)/glucose oxidase (GOD)/catalase (Cat)-modified glassy carbon electrode (GCE) in (**a**) air saturated buffer solution without (dashed line) and with glucose (black line) and (**a**,**b**) with glucose and methylviologen (MV) (blue line) and TMAO (red trace). Measurements were performed in Sörensen phosphate buffer pH 7; TorA 5.9 nmol/cm^2^, GOD 10 U, Cat 1000 U, PVA 3%. Reference electrode: Ag/AgCl/1 M KCl, Scan rate: 5 mV/s.

A TorA biosensor was prepared in which the above described oxygen scavenging membrane was used to prevent the diffusion of dissolved oxygen to the surface of a TorA-modified GC electrode. CV experiments were carried out in order to check if both the sensing layer (consisting of the adsorbed TorA) and the O_2_-scavenging membrane (with GOD and Cat entrapped in a hydrogel) led to an efficient TMAO biosensor. First, a CV was recorded in the buffer solution containing dissolved oxygen. The voltametric trace showed the typical cathodic peak attributed to the electrochemical O_2_ reduction (Figure 2a, dashed line). The reduction current decreased dramatically upon addition of glucose (Figure 2a, black line) as a result of the enzymatic removal of dissolved oxygen in the vicinity of the electrode surface. This point is consistent with the above results and similar to the background curve obtained in a nitrogen-purged solution. A comparable result was obtained with an amperometric experiment where the current in the presence of glucose was the same in anoxic conditions in an argon atmosphere. When MV was added to the buffer solution also containing also, a very clear reversible couple of peaks appeared (Figure 2a,b, blue line). This reversible CV of MV changed to a typical asymmetric catalytic CV with strong current reduction in the presence of TMAO (Figure 2b, red line). This result clearly indicates that the coupling between the immobilized oxygen-scavenging system and the Tor A-catalyzed TMAO reduction occurred.

**Figure 3:**
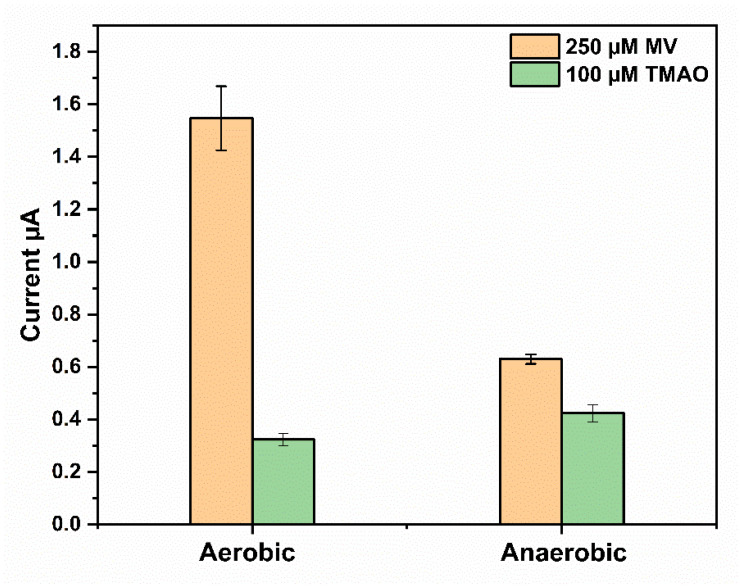
Effect of O_2_ on the response of the TMAO sensor. Measurements were performed in Sörensen buffer 67 mM pH 7; 250 µM MV and 100 µM TMAO were used in each experiment; TorA (5.9 nmol/cm^2^); GOD 10 U, Cat 1000 U, polyvinyl alcohol (PVA) 3% and without (left) and with (right) 3 mM glucose for the anaerobic conditions. (*n* = 3 electrodes, each 3 measurement).

The amperometric response of the sensor was then compared in the absence (aerobic) and presence of glucose (anaerobic), and the height of the reduction current was evaluated with 250 µM MV and 100 µM TMAO. The latter is a TMAO level found in pathological situations [5]. As shown in Figure 3, the presence of O_2_ (experiment without addition of glucose to the bulk solution) resulted in a more than two times higher MV reduction current. Higher MV reduction currents were due to an electrochemical regeneration of the MV+● that was consumed by the reaction with oxygen (Reaction 6, Scheme 1). The subsequent addition of 100 µM TMAO resulted in a sensor response of about 323.5 ± 23.1 nA. This value is 27% lower than the sensor response of about 433.77 ± 33.21 nA upon addition of the same concentration of TMAO in the absence of O_2_ (experiment with addition of glucose to the bulk solution).

2MV^+●^ + O_2_ ↔ 2 MV^2+^ +H_2_O_2_ + 2OH^−^ (6)

It should be recalled that the MV reduction and also the electrochemical reduction of oxygen produced reactive oxygen species, which may, together with the deleterious effect of molecular oxygen on the active site of TorA [20], reduce the activity of TorA. In addition, less MV+● was available for the enzyme molecules at a higher distance from the electrode surface, since the regeneration proceeds only in the vicinity of the electrode. These observations support the fact a TMAO biosensor based on TorA requires anoxic conditions to improve the sensor response to TMAO and to allow the detection of a low TMAO concentration.

#### 3.2.2. Amperometry for the Selection of a Mediator

**Figure 4:**
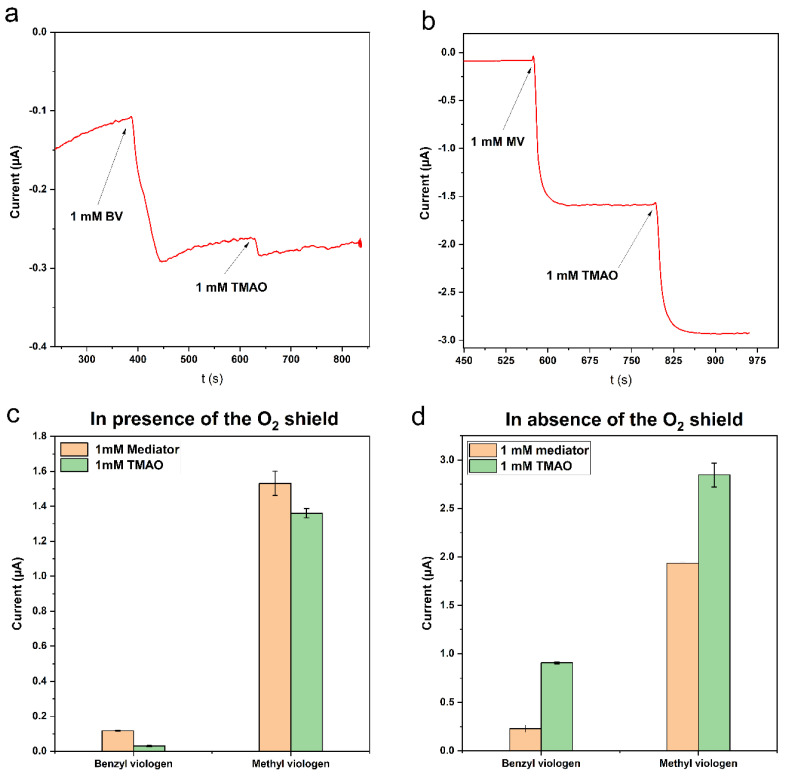
Comparison of the effect of BV and MV on TMAO biosensor response. Current response trace upon addition of 1 mM (**a**) BV and (**b**) MV followed by addition of 1 mM TMAO. Comparison of the mediator effect on the sensor response (**c**) with GOD/Cat membrane and (**d**) with GOD and Cat in solution. The TMAO biosensor used in these experiments was composed of a GCE modified with the TorA (5.9 nmol/cm^2^) and the oxygen-scavenging membrane. Measurements were performed in Sörensen buffer pH 7, 3 mM glucose. The solution was stirred during measurements (*n* = 3 electrodes, three measurements each).

Viologens are commonly used as redox indicators in enzymatic reactions catalyzed by reductases, which have a low redox potential. Previously, we determined the catalytic potential of Tor A by direct electrochemistry to be E_cat_ = −0.42 ± 0.03 vs. Ag/AgCl 1 M KCl [20] and demonstrated the electrocatalytic reduction current in the presence of TMAO and the MV mediator. In solution assays, however, Tor A is usually studied using benzyl viologen (BV) [18,19]. The choice of BV derives certainly from its lower toxicity compared to MV. Since BV was mostly used in solution, we compared its efficiency with the MV in our system. The amperometric measurements of 1 mM TMAO were carried out with the GCE previously modified with Tor A (5.9 nmol/cm^2^) and covered with the optimized O_2_-scavenging membrane under the same conditions using either BV (Figure 4a) or MV (Figure 4b) as mediator. Furthermore, the biosensor response (Figure 4c) for both mediators and TMAO was compared with the response of a TorA modified electrode and GOD and Cat in homogeneous solution (Figure 4d) in order to evaluate the influence of diffusion.

Despite the very close reported potential of MV (*E^o^ =* −0.446 V vs. NHE) [28] and BV (*E^o^* = −0.350 V vs. NHE) in aqueous media [29], higher currents were measured for MV than BV, as shown in Figure 4a,b. This behavior resulted in a low response of the sensor upon addition of TMAO in the presence of BV (Figure 4c). The sensor response (118 nA) upon addition of 1 mM BV was more than ten-fold lower than that obtained after addition of 1 mM MV (1351 nA), and the response time for BV reduction (1.75 min) was much longer compared to about 30 s for MV. The response of the TMAO biosensor for TMAO was higher when MV was used as mediator (sensor sensitivity for TMAO: 30 and 1361 nA/mM TMAO for the use of BV and MV, respectively). The very low sensitivity with BV (Figure 4c) in the presence of the O_2_-scavenging membrane can be explained by its higher molecular weight (408.11 g/mol) in comparison to that of MV (186.3 g/mol), impeding its diffusion through the dialysis membrane and the PVA membrane (O_2_ shield). Indeed, in the absence of the membrane, the response was higher for both mediators. Moreover, experiments performed in the absence of the O_2_-scavenging membrane but with soluble GOD and Cat (Figure 4d) have shown that the sensitivity of the TMAO biosensor was still higher in the presence of MV at the applied potential of −0.8 V. In addition, these results indicate that viologens can be used in solution or directly incorporated into polymers. Recently, a voltametric study of nitrogenase was performed using different types of mediators including viologens [30]. The results provided by this study have shown that viologen compounds including ethyl viologen, BV and MV were the best choice, since they showed the highest *k_cat_* in comparison to the other screened mediators.

### 3.3. Amperometric TMAO Biosensor

#### Optimization of Mediator and Immobilized TorA Concentrations

In the next step, the electrochemical TMAO biosensor was further optimized and characterized by using amperometry.

**Figure 5:**
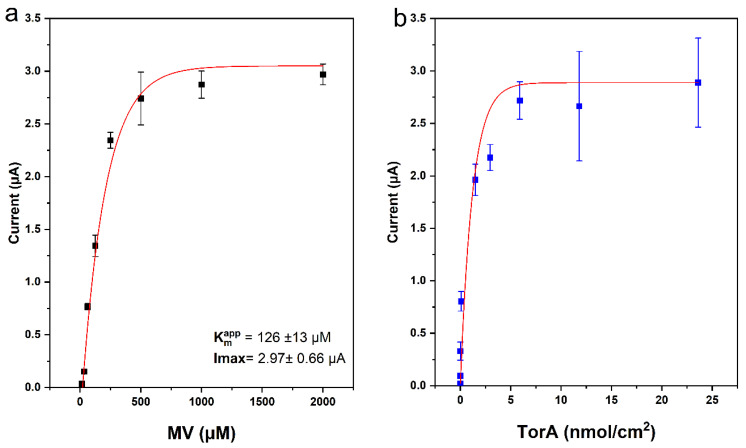
Current response of the TorA/GOD/Cat-electrode as a function of (**a**) methyl viologen concentration and (**b**) TorA surface concentration. Measurements were performed in Sörensen phosphate buffer pH 7 containing 3 mM glucose; 1 mM TMAO in (**a**) TorA 5.9 nmol/cm^2^ and in (**b**) 1 mM MV. The O_2_-scavenging membrane contained GOD 10 U, Cat 1000 U, PVA 3%, Eappl = −0.8 V, *n* = 9 (3 electrodes, three repetitions each).

To study the influence of MV concentration on the TMAO detection with the biosensor composed of immobilized Tor A and the O_2_-scavenging enzyme membrane, the concentration of MV was varied between 0.015 mM and 2 mM, and the amperometric sensor response to 1 mM TMAO was evaluated under ambient air conditions. As shown in Figure 5a, even at low concentrations of MV, the sensor responded upon addition of TMAO. An almost maximum catalytic current value was obtained after addition of 500 µM MV. This value is twice that published in our previous work [17], where already 250 µM MV were sufficient to generate a maximum current response. In the previous work the solutions where also kept oxygen-free, and therefore the parasitic reaction between MV and oxygen was omitted. The hydrogel layer (i.e., the O_2_-scavenging membrane) on top of the TorA-layer comprises an additional diffusion resistance for MV, and consequently higher mediator concentrations were needed to obtain the maximum current. Together with the slower diffusion, the typical increase of characteristic diffusion time, i.e., longer response time, was also observed, in accordance to the literature [31]. The apparent *K_m_* value for MV (@ 1 mM TMAO) for this biosensor was calculated to be 126 ± 13 µM MV. This value is comparable to an already published *K_m_* (MV) of 150 µM at pH 6.9 [32], where the *K_m_* of MV was determined for the reaction in solution. Furthermore, the hydrogel also restricts the removal of MV after solution exchange. This could be noticed when TMAO was added in a subsequent experiment with an MV-free solution, where a reduction current could be observed (even without further addition of MV). A similar approach using an MV-based polymer as a mediator and the O_2_-scavenging membrane was successfully used in a biofuel cell based on hydrogenase [33]. This interesting observation with an MV reservoir provided by the polymer suggests a way of building a reagentless single-use sensor, thus reducing the risks arising from its toxicity while adding it to the bulk solution. Here we aim at a reusable sensor and therefore provide sufficient MV-concentration to the solution.

To optimize the biosensor, the dropcasted amount of TorA was then varied between 7.02 pmol/cm^2^ and 23.6 nmol/cm^2^. The current response of the resulting biosensor upon addition of 1 mM TMAO to a buffer solution containing 1 mM MV and 3 mM glucose was characterized. As depicted in Figure 5b, the sensitivity of the sensor increased with the increasing concentration of the TorA, up to 5.9 nmol/cm^2^. Above this value the sensor response did not increase anymore, due to the diffusion limitation of the sensor response that was reached. From all the above-mentioned observations, 5.9 nmol/cm^2^ TorA and 1 mM MV were chosen as optimal concentrations for the sensor construction.

### 3.4. PH Dependency

**Figure 6:**
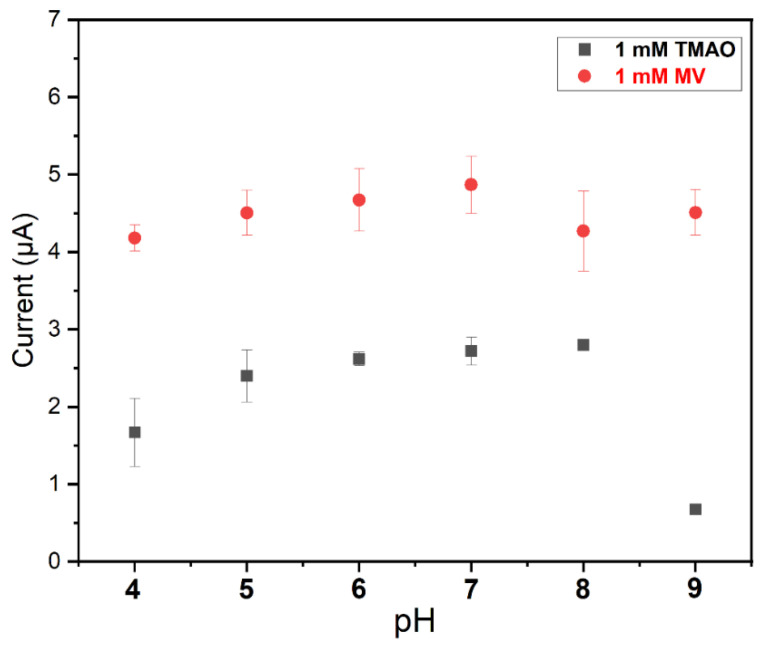
pH dependency of the response of the TorA biosensor to (red) mediator and (black) TMAO. TorA (5.9 nmol/cm^2^) GOD 10 U, Cat 1000 U, 3 mM glucose, PVA 3%. Measurements were performed in Sörensen phosphate buffer with different pH values (5, 6, 7 and 8) and containing 3 mM glucose. Solutions used to perform experiments at pH 4 and pH 9 were obtained by adding a solution of HCl (100 mM) and NaOH (100 mM), respectively, to the previous Sörensen phosphate buffer (67 mM) containing 1 M KCl (*n* = 9, 3 electrodes).

The pH dependency of the TMAO biosensor was analyzed by varying the pH of the measuring buffer solution between 4 and 9. As shown in Figure 6, the sensor presents a very broad pH optimum (between pH 5 and pH 8), with the highest values between 6 and 8. The sensor response clearly drops at a very high pH, while the MV sensitivity is the same. These values correspond fairly well to the optimum pH reported for GOD [34], Cat [35] and TorA [36] in solution, which is advantageous. This indicates that the immobilization of all these enzymes does not significantly affect their catalytic function. Moreover, the mediator reaction was not negatively impacted by the pH.

### 3.5. Biosensor Performance

**Figure 7:**
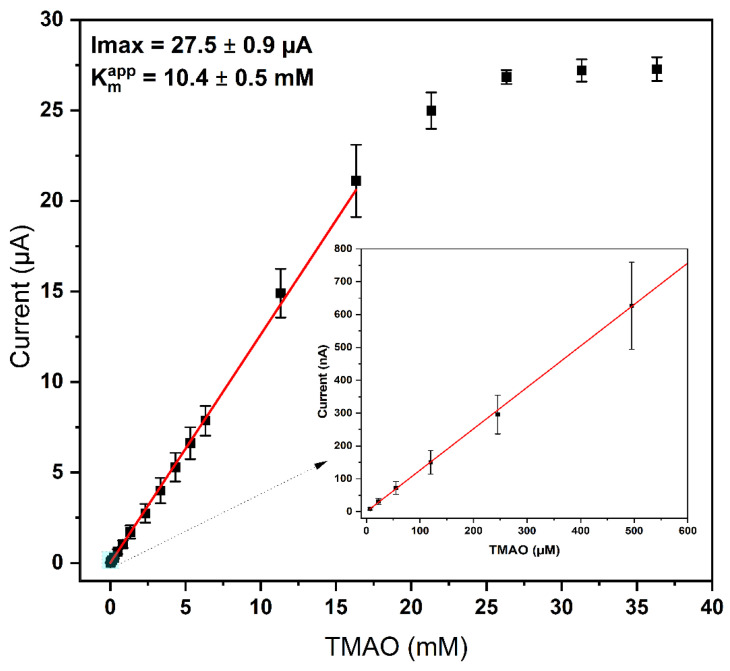
Graph of current response versus TMAO concentrations. Red line is the linear fit between 0.002 and 17 mM TMAO. The inset shows the range with low concentrations (2 µM–600 µM). Measurements were performed in Sörensen buffer pH 7; 1 mM MV was used in each experiment; TorA (5.9 nmol/cm^2^); GOD 10 U, Cat 1000 U, PVA 3% and glucose 3 mM. (*n* = 5 electrodes, 3 repetitions per concentration and electrode). Red line: Linear fit: Y = 1295.8X − 95.83; R-square = 0.999 (Range of Fitting = 0.002–16.3 mM, slope nA/mM).

The TMAO biosensor was characterized using amperometry and the above-determined optimal conditions (1 mM MV, 5.9 nmol/cm^2^ TorA, Sörensen phosphate buffer pH 7, 3 mM glucose). The addition of TMAO resulted in a fast cathodic current response plateauing after about 30 s. The response was strongly dependent on the TMAO concentration. As shown in Figure 7, the range of linearity is very broad and extends from 2 µM to about 15 mM. A linear regression of the data of the concentration dependence was made with the result Y = 1295.8X − 95.83; R-square = 0.99 (range of fitting = 0.002–16.3 mM, current in nA) (Figure 7, red line), with a lower limit of detection (LOD = 3 × sd (standard deviation)) of about 0.4 µM. In comparison to the previously published electrochemical TMAO biosensor [17], the obtained linearity range (2 µM–15 mM) of this work is comparable, despite the additional scavenger membrane. However, the response time of 33 ± 5 s is twice as high, and the sensor sensitivity is two times lower in comparison to our earlier work [17], which can again be attributed to the additional diffusion resistance. This observation was also confirmed by the loading experiments, whereby more MV and TMAO were needed in comparison to a system without membrane. Improvements of the O_2_-scavenging membrane’s permeability might also help to decrease the response time of the sensor, since the obtained values also depend on the thickness of the layers present on top of the electrode [37]. From 10 repetitive measurements of 100 µM TMAO on several days, the coefficient of variation and standard error of the mean of ≤7% and ≤3.44 were calculated. These values are also comparable to the value of 8.8% calculated for the biosensor with soluble-oxygen-scavenging enzymes [17]. A previously reported imprinted-polymer-based TMAO sensor needs 20 min per measurement and has a linear range of 1–15 ppm (13–200 µM TMAO) [16]. See Table S1 (supporting information).

**Figure 8:**
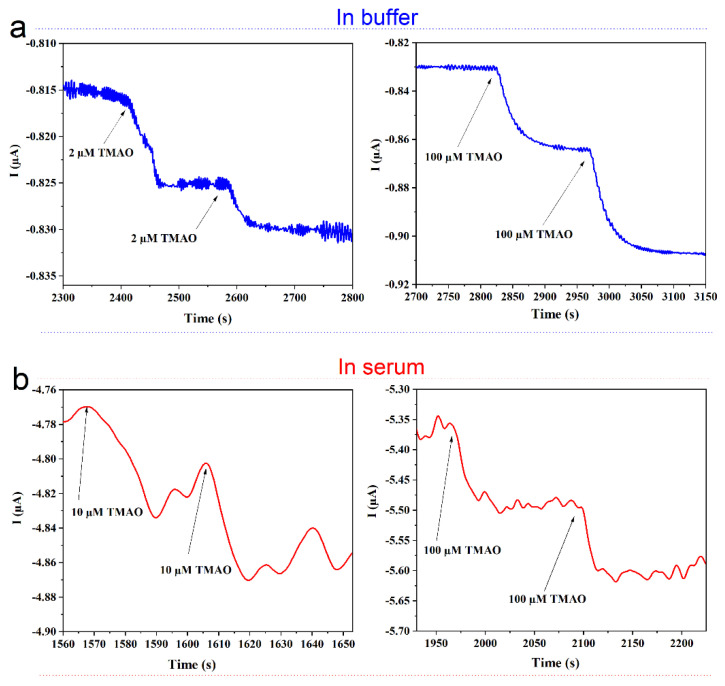
TMAO detection with the optimized TMAO biosensor. Amperometric detection of different concentrations of TMAO (**a**) in buffer and (**b**) in 1:10 diluted serum in buffer. The buffer was a Sörensen phosphate buffer 67 mM pH 7 containing 1 mM MV and 3 mM glucose; the electrode was modified with TorA (5.9 nmol/cm^2^); GOD 10 U; Cat 1000 U; PVA 3%.

The biosensor was thus applied to measurements in serum samples spiked with different concentrations of TMAO. To adjust the pH and due to the addition of MV, the experiments were conducted in a slightly diluted serum. In Figure 8, amperometric measurements of TMAO in buffer (a) and in (10-fold diluted) serum (b) are compared. A clear increase of current is observed upon addition of TMAO in the buffer (Figure 8a) and in the serum (Figure 8b). The addition of 2 µM TMAO to the buffer solution is clearly distinguishable, while the noise of the background in the diluted serum is so high that the lowest measurable concentration of TMAO was 10 µM TMAO. From the standard deviation (sd), the lower limit of detection (LOD = 3 sd) of 3.3 µM was calculated for the measurement of TMAO in the serum solution. The sensitivity of the biosensor was higher in the buffer in comparison to the diluted serum, as the serum induced an increase of the noise probably due to an unspecific interaction with the dialysis membrane [38]. It must be noted that in our previous work, where the bulk solution was enzymatically deoxygenated by the GOD and Cat directly in solution, no negative effect of the serum was observed [17]. These observations indicate that the serum unspecifically interacts at the membrane surface, thus affecting the diffusion of either or both the mediator and TMAO. It was reported that the TMAO level in the blood of healthy humans is between 2.25 and 5.79 µM, whereas patients with CVDs and associated diseases have substantially higher levels in their blood (up to 100 µM) [39]. Thus, the sensor is not yet sufficiently sensitive to reach the threshold value for high risk, but can be used to detect the elevated TMAO level in case of, e.g., a chronic kidney disease.

### 3.6. Sensor Stability

**Figure 9:**
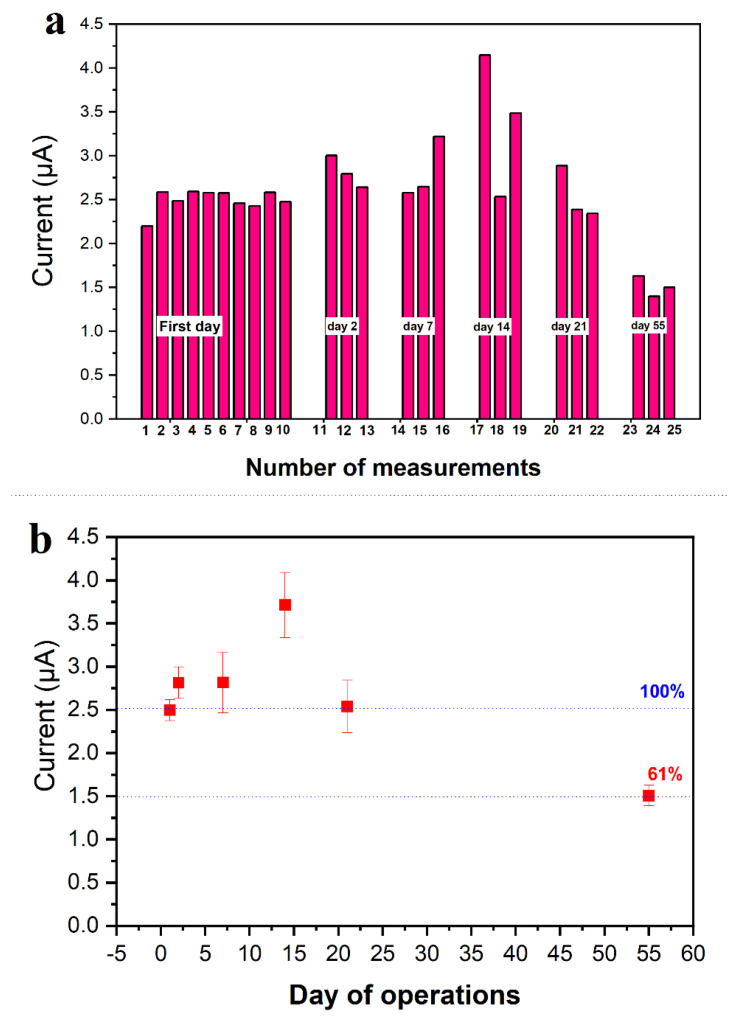
Stability of the TMAO biosensor. Current response for repetitive measurements with TorA-biosensor conducted during 2 months with the same sensor. (**a**) Current response of each measurement. (**b**) Average of the obtained current of each measurement day. Measurements were performed in Sörensen phosphate buffer pH 7 containing 1 mM MV and 3 mM glucose; addition of 1 mM TMAO and in each measurement; electrode modified with TorA (5.9 nmol/cm^2^) covered by a membrane of GOx 10 U, Cat 1000 U, PVA 3%.

The stability of the developed sensor was followed over a period of 2 months. To achieve this goal, the same electrode was challenged with 0.1 and 1 mM TMAO 10 times on the first day of sensor use, and then always three times in the following days and weeks (Figure 9a). The precision and the repeatability of the sensor was tested in the first day of operation, whereby a coefficient of variation of about 6.2% and 4.6% was calculated for 10 successive measurements of 0.1 mM and 1 mM TMAO, respectively. Moreover, the oxygen shield was also stable over 3 weeks, since additions of glucose resulted always in a complete O_2_ depletion. The sensor sensitivity did not decrease within the first three weeks and dropped only 40% after 2 months (Figure 9b). A slight increase of the TMAO sensitivity of the biosensor was observed during the first 2 weeks. This observation might be accounted for by the progressive swelling process of the O_2_ shield. The swelling of the membrane might result in a better diffusion for TMAO and MV, thus improving the response of the biosensor upon addition of the TMAO. However, the reduced sensitivity at the end of the tested period no longer allowed for the indication of the low TMAO level of healthy people. In our previous work [17], an activity loss of about 12% after 1 week was observed. These observations indicate a stabilizing effect of the O_2_-scavenging membrane on the new TMAO biosensor.

## 4. Conclusions

In this work, a new electrochemical TMAO biosensor based on TorA was developed. In order to perform measurements without O_2_ interference, an O_2_-scavenging membrane based on a bienzymatic reaction was constructed using PVA hydrogel. The applied bi-enzyme membrane is efficient for locally protecting the sensing layer of the TMAO biosensor from dissolved O_2_. This enables enzymatic TMAO measurements without specific laboratory equipment to establish an oxygen free environment. Analytical applications and TMAO measurement will be much simplified, as has been proposed for several other biosensors [21,24,27], including a DMSO biosensor [25]. Hence, this system can be envisaged for future use with other reductases. Moreover, the sensor performance in the configuration based on GCE with an enzyme variant that is much easier to produce was comparable to the recently published results by Mitrova and coworkers using an engineered variant of the enzyme TorA [17] combined with the needed consumption of a (soluble) enzyme for each measurement. Although the additional layer (O_2_ shield) reduces the sensitivity and increases the response time of the electrochemical biosensor, the obtained performance still allows for the detection of as low as 2 µM TMAO in a plain buffer solution with a LOD of 0.4 µM. This limit of detection using a GCE modified with both an O_2_-scavenging membrane and TorA remains acceptable for the monitoring of healthy and unhealthy TMAO levels in humans. With the addition of 10 µM of diluted serum, TMAO could be followed. This could indicate the appearance of unhealthy levels of TMAO, which may reach values of 100 µM [39]. Up to now, the procedure involves a dilution step when the sample is added to the measuring cell, and therefore, in the present state of development, the achieved sensitivity is not enough to reach an analytically interesting concentration in undiluted real samples. Further improvements can be envisaged by new polymers which can enhance the diffusion of the analyte. Moreover, immobilized viologens may also be introduced, as was previously shown for other target molecules [33]. Moreover, using a methyl-viologen-loaded polymer might prevent the waste of toxic agents and thus the applicability of the sensor approach. Progress in these directions will be the scope of a future report. Finally, the developed sensor reported here represents a step forward for the development of a sensor device for screening of elevated TMAO values in human blood.

## Data Availability

Not applicable.

## Supplementary Materials

The following are available online at https://www.mdpi.com/article/10.3390/bios11040098/s1. Figure S1: Glucose oxidase reaction on Clark electrode. Cyclic voltametric measurements of an electrode covered with GOD immobilized in PVA (3%) with serial addition of glucose (0–6 mM) (a). Amperometric detection of the oxygen consumption at −0.6 V (b) and hydrogen peroxide production at +0.6 V (c) upon addition of glucose with a membrane including GOD entrapped in 3% PVA hydrogel. (d) Optimization of the GOD present in the oxygen-scavenging membrane in order to get a stable anoxic condition. All these measurements were performed in a Sörensen phosphate buffer (pH 7) containing 100 mM KCl under ambient air conditions. Figure S2: Current trace of a modified glassy carbon electrode on the serial addition of 0.3 mM glucose. The inset represents the current change vs. glucose concentration. Measurements were performed in a Sörensen buffer pH 7; aerobic condition. The oxygen shield consisting of GOD 10 U, Cat 1000 U, PVA 3% was immobilized on a glassy carbon electrode. Applied potential Eappl = −0.8 V vs. Ag/AgCl, 1 M KCl. Table S1: Comparison of TMAO sensors.
