# Supplementary material for: Electrochemical Trimethylamine N-Oxide Biosensor with Enzyme-Based Oxygen-Scavenging Membrane for Long-Term Operation under Ambient Air
Source: Biosensors (Basel). 2021 Mar 27;11(4):98. doi: 10.3390/bios11040098 (PMC8066520; doi:10.3390/bios11040098)
Supplement: Supplementary file 1 [file biosensors-11-00098-s001.pdf]

biosensors

MDPI

Supplementary materials

Electrochemical Trimethylamine *N*-Oxide Biosensor with Enzyme-Based Oxygen-Scavenging Membrane for Long-Term Operation under Ambient Air

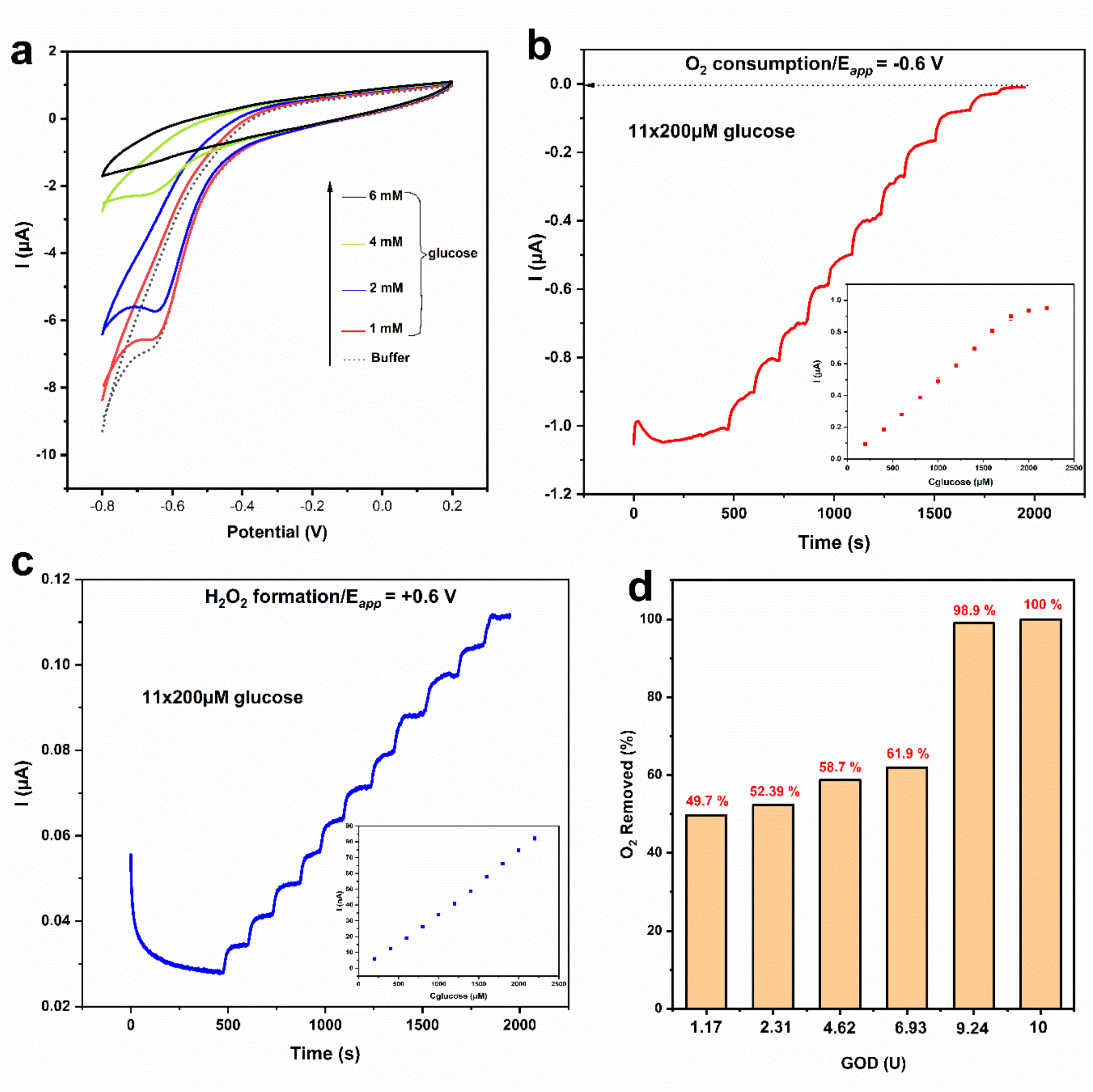

Figure S1. Glucose oxidase reaction on Clark electrode. Cyclic voltammetric measurements of an electrode covered with GOD immobilized in PVA (3%) with serial addition of glucose (0-6 mM) (a). Amperometric detection of the oxygen consumption at -0.6 V (b) and hydrogen peroxide production at +0.6 V (c) upon addition of glucose with a membrane including GOD entrapped in 3%. PVA hydrogel. (d) Optimization of the GOD present in the oxygen scavenging membrane in order to get a stable anoxic condition. All these measurements were performed in Sörensen phosphate buffer (pH 7) containing 100 mM KCl under ambient air conditions.

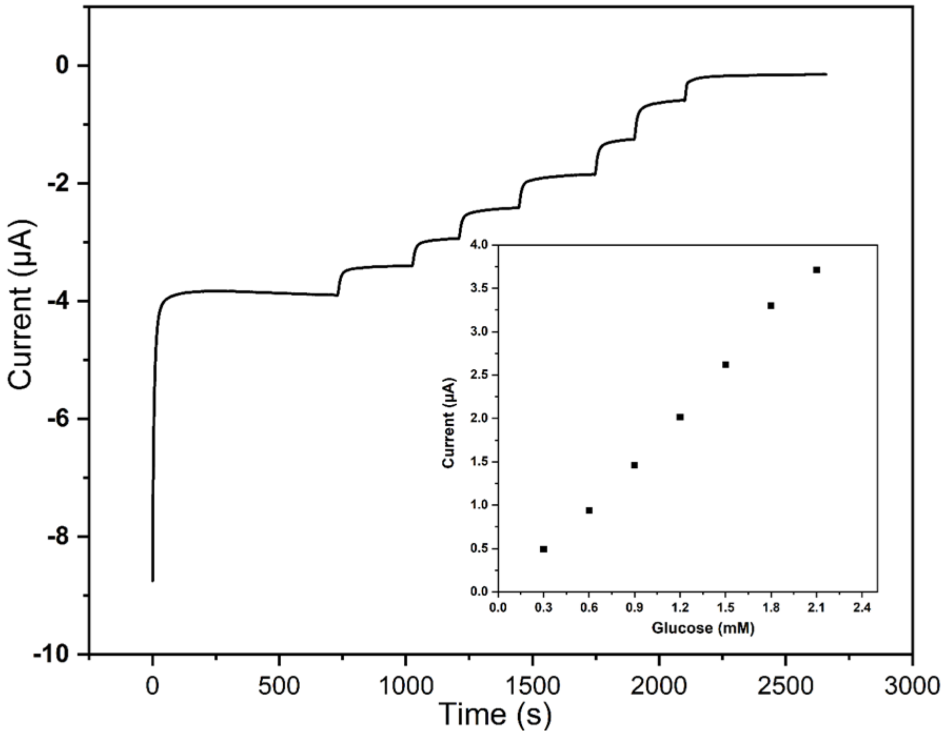

Figure S2. Current trace of a modified glassy carbon electrode on serial addition of 0.3 mM glucose. The inset represents the current change vs glucose concentration. Measurements were performed in Sörensen buffer pH 7; aerobic condition. The oxygen shield consisting of GOD 10 U, Cat 1000 U, PVA 3% was immobilized on glassy carbon electrode. Applied potential Eappl= -0.8V vs Ag/AgCl, 1M KCl.

Table S1. Comparison of TMAO-sensors.

Technique	Electrode	Linear range	LOD *	Sensitivity	Stability	Response time
Amperometry [17]	GCE	2-110µM	2,96 nM (buffer / spiked serum)	14.16 nA/µM	1 week	16 ± 2 s
DPV [16]	MIP/ITO	13-200 µM	20 µM	nr	nr	16 min
Amperometry (present work)	GCE	2-15000 µM	0.4 µM (buffer)	2.75 nA/µM	3 weeks	33 ± 5s

*calculated from 3 x SD of the blank, or small TMAO concentration, resp., GC: glassy carbon electrode, MIP: molecular imprinted polymer, ITO: indium doped tin oxide; nr: not reported, DPV: differential pulse voltammetry.
